# Retinal Targets ALDH Positive Cancer Stem Cell and Alters the Phenotype of Highly Metastatic Osteosarcoma Cells

**DOI:** 10.1155/2015/784954

**Published:** 2015-12-24

**Authors:** Xiaodong Mu, Stuti Patel, Damel Mektepbayeva, Adel Mahjoub, Johnny Huard, Kurt Weiss

**Affiliations:** ^1^Department of Orthopaedic Surgery, University of Texas Health Science Center at Houston, Houston, TX 77030, USA; ^2^Cancer Stem Cell Laboratory, Department of Orthopaedic Surgery, University of Pittsburgh, Pittsburgh, PA 15219, Kazakhstan; ^3^Laboratory of Cellular Technology, Nazarbayev University, Astana, Akmola 010000, USA

## Abstract

Aldehyde dehydrogenase (ALDH) is a cancer stem cell marker. Retinoic acid has antitumor properties, including the induction of apoptosis and inhibition of proliferation. Retinal, the precursor of retinoic acid, can be oxidized to retinoic acid by dehydrogenases, including ALDH. We hypothesized that retinal could potentially be transformed to retinoic acid with higher efficiency by cancer stem cells, due to the higher ALDH activity. We previously observed that ALDH activity is greater in highly metastatic K7M2 osteosarcoma (OS) cells than in nonmetastatic K12 OS cells. We also demonstrated that ALDH activity correlates with clinical metastases in bone sarcoma patients, suggesting that ALDH may be a therapeutic target specific to cells with high metastatic potential. Our current results demonstrated that retinal preferentially affected the phenotypes of ALDH-high K7M2 cells in contrast to ALDH-low K12 cells, which could be mediated by the more efficient transformation of retinal to retinoic acid by ALDH in K7M2 cells. Retinal treatment of highly metastatic K7M2 cells decreased their proliferation, invasion capacity, and resistance to oxidative stress. Retinal altered the expression of metastasis-related genes. These observations indicate that retinal may be used to specifically target metastatic cancer stem cells in OS.

## 1. Introduction

Retinoids are a class of compounds comprised of several signaling molecules, such as retinoic acid and retinaldehyde, that are structurally related to vitamin A (retinol) [1]. These molecules play a crucial role in the regulation of various cellular processes. Retinoids have been shown to exert a tumor-suppressive effect on cells based on their interaction with cyclins and cyclin-dependent kinases (CDKs) to prevent cell-cycle progression [1]. Additionally, they also influence cancer cell differentiation and apoptosis [2]. Many cancers have been shown to have abnormally low levels of various retinoids.

Retinoic acid, a derivative of retinol, has been shown to inhibit proliferation of malignant tumors and induce the apoptosis and differentiation in various types of cancer cells [1, 3–6]. Due to retinoic acid's antitumor properties, its role is being investigated in the treatment of various malignancies [3]. It is currently used in the treatment of acute promyelocytic leukemia and has been shown to result in complete remission [7]. Retinoic acid is derived from its precursor, all-trans-retinal (also called retinaldehyde or vitamin A aldehyde), by the action of dehydrogenases, including aldehyde dehydrogenase (ALDH) [8]. Previous studies have demonstrated that high ALDH activity predicted poor survival in various types of cancers [9, 10], such as breast cancer [11], epithelial cancer [12], and sarcoma [13]. ALDH activity is often specifically upregulated in cancer stem cells and has been recognized as a marker for cancer stem cells [10, 14–16]. Therefore, we believe it is reasonable to hypothesize that, because of the higher ALDH activity in cancer stem cells, retinal could be more efficiently transformed to retinoic acid by cancer stem cells, and thus preferentially induce the apoptosis and alteration of these cells.

Osteosarcoma (OS) is the most common primary tumor of the bone. It has a bimodal age distribution with a major peak during the second decade of life and a second smaller peak observed in patients over 50 years old. The lung is the most common site of metastatic spread, and survival is ultimately determined by the presence of absence of pulmonary metastatic disease. Deaths related to OS are thus the result of metastasis to the lung rather than the primary tumor itself. The prognosis of patients with OS has not improved in the past several decades due to the lack of treatment specifically targeting OS cell metastatic potential. Due to our limited understanding of the biology of sarcoma metastasis, this problem remains unsolved.

We have demonstrated the role of various cytokines and signaling pathways in OS metastasis using two related cell populations of a spontaneously occurring murine OS [17–20]. These cell lines, K12 and K7M2, differ in their metastatic potential, with K7M2 showing more features of cancer stem cells and a much greater metastatic rate to the lungs compared to K12 cells. The difference in their characteristics and metastatic potentials demonstrated that K7M2 and K12 feature different activation statuses of multiple biologic factors and signaling pathways: ALDH, Notch1, and* *mammalian target of rapamycin (mTOR). The inhibition of these signaling pathways alters OS cell behavior* in vitro* [17–20]. We observed that K7M2 cells demonstrated greater ALDH gene expression and activity than the less metastatic K12 cells. Additionally, ALDH activity was found to be greater in cultured cells from bone sarcoma patients who experienced clinical metastasis [21]. We thus hypothesized that ALDH might represent a means to specifically target sarcoma cells with high metastatic potential.

Our current research investigates the effect of novel treatment approaches to these metastasis-related genes. In the present study, we sought to take advantage of the increased level of ALDH activity in highly metastatic K7M2 OS cells to study the effects of retinal on cell growth and metastasis. Given the ability of retinal to be transformed into retinoic acid by ALDH and the high level of expression of ALDH in cancer stem cells, we hypothesized that treatment with retinal would selectively induce apoptosis and suppress tumor growth in K7M2 OS cells.

## 2. Materials and Methods

### 2.1. Cell Culture and Retinal Treatment

K12 and K7M2 cells were cultured in proliferation medium (10% FBS in DMEM). 5,000 cells were seeded in each well of a 12-well plastic plate. Cells were treated with either 1 *µ*g/mL or 5 *µ*g/mL of all-trans-retinal (Sigma) dissolved in 1 mL of proliferation medium. K7M2 cells treated with 1 *µ*L of DMSO served as control. Cells were incubated for 2 days, before being fixed for observation or harvested for mRNA isolation.

### 2.2. Fluorescence-Activated Cell Sorting (FACS) Analysis of ALDH Activity and Sorting of Cells

ALDH enzymatic activity was determined using the Aldefluor Kit (STEMCELL Technologies). Cultured K7M2 cells from treatment as well as control groups were resuspended in Aldefluor buffer (1 × 10^6^ cells/mL) and incubated at 37°C as per manufacturer's instructions. During the cell sorting process, cells were washed in Aldefluor buffer and maintained at 4°C. FL1 channel of a BD FACSAria Cell Sorting System and FACSDiva software (version 6.1.2; Becton, Dickinson and Company, San Jose, CA) was used to assess ALDH activity. Fluorescence-activated cell sorting (FACS) was utilized to collect cells according to their fluorescence intensity, which corresponds to cells' ALDH activity level. Cells with high and low ALDH levels were separately isolated and cultured.

### 2.3. Semiquantitative Reverse Transcription Polymerase Chain Reaction (RT-PCR)

RNeasy plus mini kit (Qiagen) and the iScript cDNA Synthesis kit (Bio-Rad) were used to extract RNA from the cells and generate cDNA, respectively. RT-PCR was performed using a Bio-Rad MyiQ thermal cycler (Bio-Rad).  Table 1 shows the sense and antisense primers for RT-PCR and their product. Following cycling parameters were used for all reactions: 94°C for 5 minutes; 30 cycles of the following: denature for 45 seconds at 95°C, anneal for 30 seconds (53°C–56°C), and extend for 45 seconds at 72°C. Expression of target genes was normalized to the expression of GAPDH, which served as a control gene. ImageJ software (version 1.32j, National Institutes of Health, Bethesda, MD) was used to perform RT-PCR analysis. The integrated density of bands was calculated based on product of the area and the mean gray value. All molecular bands were normalized to GAPDH.

### 2.4. Cell* In Vitro* Invasion Assay

*In vitro *invasion capacity of K7M2 cells treated with retinal was compared to control cells using a real-time cell invasion and migration (RT-CIM) assay system (ACEA Biosciences, Inc.), with a 16-well transwell plate (CIM-plate 16, Roche Diagnostics GmbH). The surface of the wells in the upper chamber was coated with Matrigel (BD BioSciences, Bedford, MA, USA) of different concentrations (2.5%, 5%, and 10%). Serum-containing medium (10% FBS) was added to the wells of the lower chamber. Cells (4 × 10^4^ per well) in serum-free medium were seeded in the upper chamber. The migration of the cells through the Matrigel was monitored by the system every 15 minutes for 24 hours. Data analysis was carried out using RTCA Software 1.2 supplied with the instrument.

### 2.5. Actin Staining

Cells from each group were treated with phalloidin conjugated with Alexa Fluor 488 (Invitrogen) to observe actin organization. Treatment and control cells were grown overnight in proliferation medium in 12-well plates (50,000 cells/plate). The following day, the plates were washed twice with PBS, fixed in 3.7% formaldehyde solution for 10 minutes at room temperature, and washed two more times with PBS. The cells were then permeabilized in 0.1% Trition X-100 for 20 minutes and washed again with PBS. 5 *μ*L of methanolic stock solution phalloidin, 200 *μ*L PBS, and 1% BSA were added to each well and allowed to stand for 20 minutes, before being washed again with PBS. 300 *μ*L of 300 nM DAPI was used for nuclear staining.

### 2.6. Statistical Analysis

Statistical analysis of all experiments for this study was carried out using data from at least three samples from each treatment group. Statistical significance was evaluated using Student's *t*-test. Difference between groups was considered significant for *p* value <0.05.

## 3. Results

### 3.1. Retinal Treatment of Highly Metastatic K7M2 Cells Decreased the Proliferation Capacity and Resistance to Oxidative Stress

**Figure 1:**
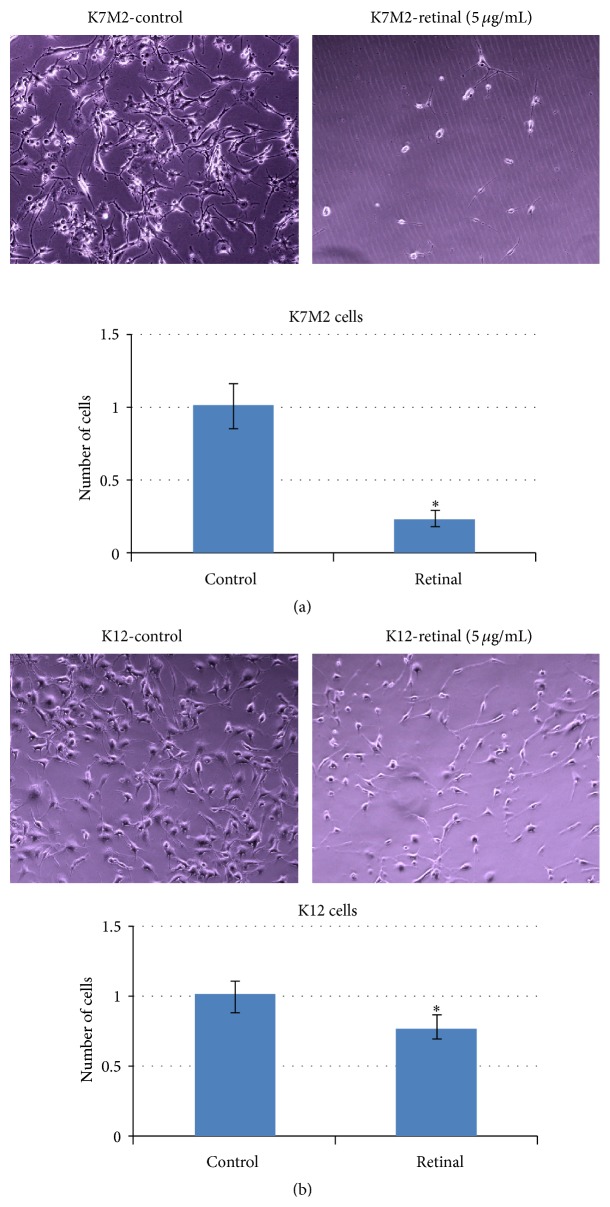
Retinal treatment of K7M2 cells decreased cell proliferation or survival. (a) and (b) The cell proliferation or survival capacity of K7M2 cells was more reduced with retinal treatment (5 *µ*g/mL, 2 days), compared to K12 cells.

**Figure 2:**
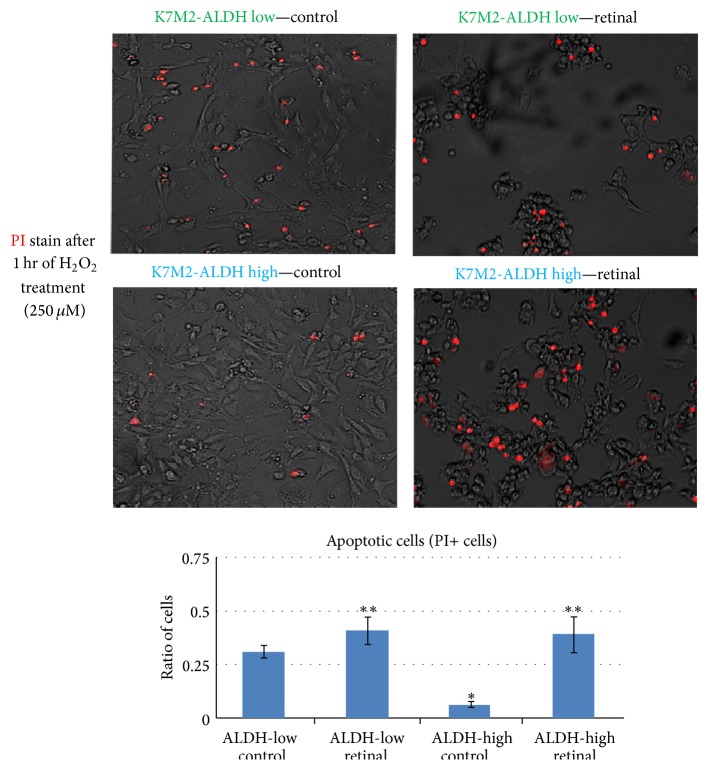
Retinal treatment of sorted ALDH-high K7M2 cells decreased antioxidative stress capacity. After cell sorting according to ALDH activity, it showed that the K7M2-ALDH high cells were more resistant to oxidative stress (H_2_O_2_, 250 *µ*M for 1 hr) than the K7M2-ALDH low cells, while the retinal treatment of K7M2-ALDH high cells was able to greatly decrease their resistance to oxidative stress and resulted in more cell apoptosis. “*∗*” indicates that ALDH-high control cells are significant different (*p* < 0.05) compared to ALDH-low control cells; “*∗∗*” indicates retinal treated ALDH-high or ALDH-low cells are significant different (*p* < 0.05) compared to nontreated ALDH-high or ALDH-low control cells, respectively.

K7M2 cells have been shown to feature much higher ALDH activity compared with K12 cells [17, 18]. K7M2 cells treated with all-trans-retinal (5 *µ*g/mL) for 2 days were observed to have fewer cells on the culture dishes compared with the nontreated K7M2 cells (Figure 1(a)). Actually, compared to retinal-treated K12 cells, the proliferation of retinal-treated K7M2 cells was much more obviously decreased (Figure 1), indicating preferentially increased apoptosis in retinal-treated K7M2 cells. Furthermore, retinal-treated K7M2 cells also displayed higher levels of apoptosis induced by exposure to hydrogen peroxide (250 *µ*M, 1 hr), as seen by propidium iodide (PI) staining (data not shown). Our previous results have shown that the sorted ALDH-high K7M2 cells are more resistant to oxidative stress-induced cell death, compared with the sorted ALDH-low cells [17]. Treatment of ALDH-high K7M2 cells with retinal (5 *µ*g/mL) for 2 days significantly increased the ratio of apoptotic cells induced by exposure to hydrogen peroxide, compared to nontreated control ALDH-high K7M2 cells (Figure 2). The apoptotic effect was much more pronounced than in ALDH-low K7M2 cells. This observation indicates that the resistance to oxidative stress of K7M2 cells was preferentially decreased by retinal in cells with high ALDH activity.

### 3.2. Retinal Treatment of K7M2 Cells Resulted in Modified Gene Expression of Metastasis-Related Factors and Reduced ALDH Activity

**Figure 3:**
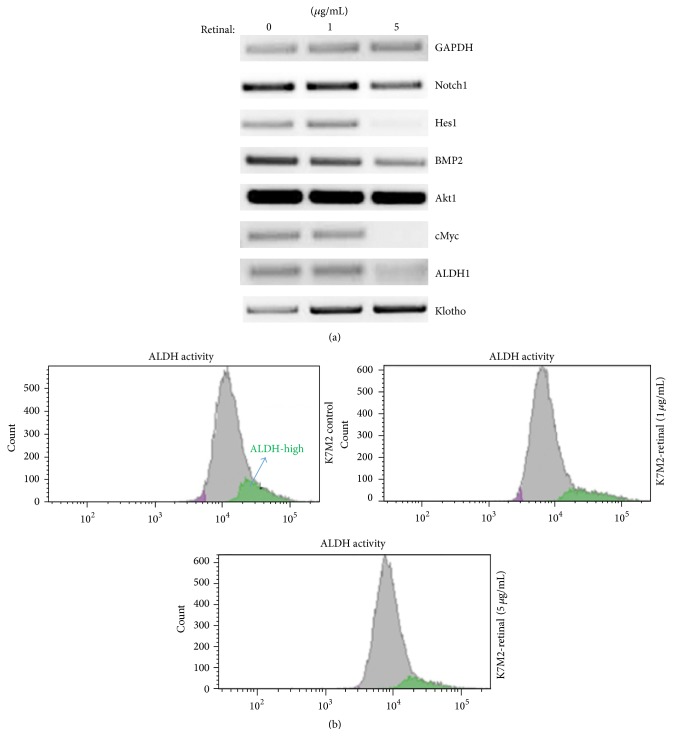
Retinal treatment of K7M2 cells modified the expression of metastasis-related genes and reduced ALDH activity. (a) RT-PCR results showed retinal treatment of K7M2 cells (1 or 5 *µ*g/mL for 2 days) modified the expression of some key genes related with metastasis or tumorigenesis. (b) ALDH activity assay showed that retinal treatment of K7M2 cells reduced the aLDH activity of the cells.

Semiquantitative PCR analysis of K7M2 cells with and without retinal treatment was carried out to compare the expression levels of various signaling molecules involved in cell proliferation, apoptosis, and metastasis. Treatment with retinal resulted in the downregulation of Notch signaling as evidenced by decreased expression of Notch1 and Hes1. Further, there was also a decrease in bone morphogenetic protein 2 (BMP2), cMyc, and ALDH levels while Klotho, a tumor suppressor, was up-egulated with retinal treatment (Figure 3(a)). Additionally, ALDH activity assay of K7M2 cells verified that retinal treatment (both 1 *µ*g/mL and 5 *µ*g/mL) was able to reduce the ALDH activity of K7M2 cells (Figure 3(b)).

### 3.3. Retinal Treatment of K7M2 Cells Altered Cell Morphological Characteristics and Decreased Invasion Capacity

**Figure 4:**
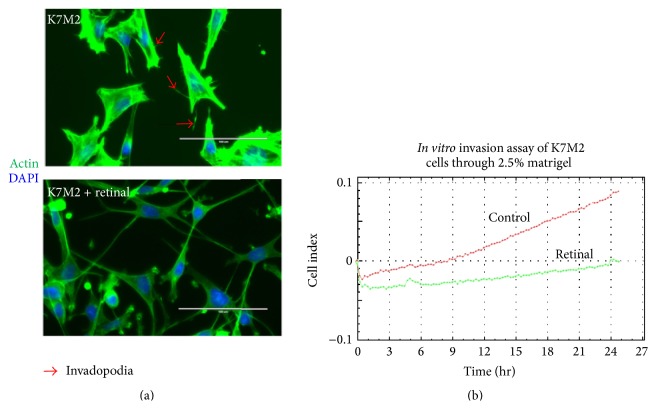
Retinal treatment of K7M2 cells changed migration-related cell morphological characteristics and decreased migration capacity. (a) Actin staining with phalloidin revealed that the cell morphology of K7M2 cells was modified with retinal treatment (5 *µ*g/mL for 2 days), showing reduced presence of invadopodia. (b)* In vitro* invasion assay with 3D-matrigel (2.5%) revealed that K7M2 cells treated with retinal treatment (5 *µ*g/mL for 2 days) migrated slower than nontreated K7M2 control cells.

Our previous results demonstrated that K7M2 cells feature different cell morphology than K12 cells and have significantly higher migration and invasion capacities* in vitro* [17, 18]. Here, actin staining by phalloidin demonstrated that K7M2 cells treated with retinal had undergone obvious alterations in migration-related cell morphological characteristics with reduced appearance of invadopodia, which play critical roles in cell migration/invasion (Figure 4(a)). Finally,* in vitro* invasion assays further displayed that treatment of K7M2 cells with retinal (5 *µ*g/mL, 2 days) resulted in decreased cell invasion compared with untreated cells (Figure 4(b)).

## 4. Discussion

All-trans-retinal is a precursor of retinoic acid and can be transformed into retinoic acid by dehydrogenases, including ALDH. Retinoic acid has been shown to induce differentiation of hematopoietic and neural stem cells. Retinoic acid appears to induce apoptosis and differentiation in a number of cancers and has been successfully used to treat acute promyelocytic leukemia [1, 7]. However, the role of retinal as an antiproliferative agent in OS has not been studied. Based on the fact that retinal can be transformed to retinoic acid by ALDH, and ALDH activity is typically higher in cancer stem cells [9, 15, 18], we investigated the effect that retinal would have on highly metastatic OS cells.

Our study revealed that retinal was able to preferentially target the highly metastatic K7M2 cells which feature greater ALDH activity and other stem cell characteristics, by repressing their proliferation and inducing the apoptosis of these cells. The role of retinal in decreasing cell proliferation and cell survival was demonstrated by exposing cells to oxidative stress using hydrogen peroxide. ALDH-high K7M2 cells exhibited a greater increase in apoptosis compared to ALDH-low K7M2 cells. Additionally, our RT-PCR results also showed that retinal treatment results in the downregulation of various genes involved in cell-proliferation and cell survival. Namely, the expression level of Notch1, Hes1, cMyc, and BMP2 was decreased in a dose-dependent manner. Further, there was an upregulation of Klotho, a tumor suppressor gene.

Additionally, use of retinal as a therapeutic agent not only employs the antiproliferative properties of retinoic acid but also takes advantage of certain factors produced by tumor stem cells specifically. The action of retinal on the cells is mediated by retinoic acid [2]. Given that the conversion of retinal to retinoic acid requires the action of dehydrogenases, it can be expected that the activity of retinal will be higher in tumor stem cells, which produce much higher quantities of ALDH, thereby making retinal therapy highly specific. Our results, therefore, indicate that retinal may be used as a cellular “Trojan Horse” of sorts to specifically target cancer stem cells.

Since pulmonary metastasis is the major cause of mortality in OS patients, it is crucial to devise therapeutic options that specifically target the metastatic potential of OS cells. Actin staining was performed to characterize the changes in cell morphology following treatment with retinal. We have previously demonstrated that changes in K7M2 cell shape and appearance of invadopodia relate to alteration in cells' migration/invasion potentials [17–21]. Our results demonstrate that in addition to acting as an antiproliferative agent, retinal also decreased cell invasiveness. Based on these results, we conclude that retinal therapy has the potential to specifically target metastatic OS cells and thus potentially decrease the incidence of pulmonary metastases, the ultimate cause of mortality in OS.

One of the limitations of this study is that the results are based on experiments carried out* in vitro*. While several studies have verified that the behavior of K7M2 cells parallels the growth of human OS cells, it is essential to conduct* in vivo* studies in order to validate these results.

## 5. Conclusion

Our current results demonstrate that retinal was capable of specifically acting on OS cells which express high levels of ALDH and promoted these cells to undergo apoptosis. As cancer stem cells typically feature high ALDH activity, retinal could be used to specifically repress the growth of cancer stem cells with enhanced metastatic potential. Our study is the first to demonstrate the potential application of retinal for the treatment of tumorigenesis. We suggest that retinal could be potentially used to target OS cells with stem-like properties and ultimately decrease the ability of these cells to metastasize.

## Figures and Tables

**Table 1:** Primer sequences.

Gene	Primer sequence
GAPDH	Forward: TCCATGACAACTTTGGCATTG
	Reverse: TCACGCCACAGCTTTCCA

Notch1	Forward: GCCGCAAGAGGCTTGAGAT
	Reverse: GGAGTCCTGGCATCGTTGG

Hes1	Forward: CCAGCCAGTGTCAACACGA
	Reverse: AATGCCGGGAGCTATCTTTCT

BMP2	Forward: TCTTCCGGGAACAGATACAGG
	Reverse: TGGTGTCCAATAGTCTGGTCA

Akt1	Forward: ATGAACGACGTAGCCATTGTG
	Reverse: TTGTAGCCAATAAAGGTGCCAT

cMyc	Forward: TGACCTAACTCGAGGAGGAGCTGGAATC
	Reverse: AAGTTTGAGGCAGTTAAAATTATGGCTGAAGC

ALDH1	Forward: GACAGGCTTTCCAGATTGGCTC
	Reverse: AAGACTTTCCCACCATTGAGTGC

Klotho	Forward: CCCAAACCATCTATGAAAC
	Reverse: CTACCGTATTCTATGCCTTC

## Abbreviations

ALDH: Aldehyde dehydrogenase
retinal: All-trans-retinal
FACS: Fluorescence-activated cell sorting
BMP: Bone morphogenetic protein
mTOR: Mammalian target of rapamycin.
